# Citric Acid-Esterified Ginger Starch: Preparation, Characterization, and Construction of Pickering High Internal Phase Emulsions Synergistically Stabilized with β-Cyclodextrin

**DOI:** 10.3390/foods15111916

**Published:** 2026-05-29

**Authors:** Xiaohong Ge, Yaru Pan, Xiaofan Lv, Haoyuan Guo, Benguo Liu

**Affiliations:** 1 School of Food Science, Henan Institute of Science and Technology, Xinxiang 453003, China; gexh801210@163.com (X.G.); 18803645210@163.com (Y.P.); lvxiaofan06@163.com (X.L.); 2 College of Food and Bioengineering, Henan University of Science and Technology, Luoyang 471023, China; 15003730801@163.com

**Keywords:** ginger starch, citric acid esterification, characterization, Pickering emulsion

## Abstract

In this study, citric acid-esterified ginger starches with different esterification degrees (EGSC10, EGSC20, and EGSC30) were prepared via a dry-heat method by adjusting the dosage of citric acid (10%, 20%, 30%, *w*/*w*). Their multi-scale structures were systematically characterized, and Pickering high internal phase emulsions (Pickering HIPEs) synergistically stabilized by EGSC30 and β-cyclodextrin (β-CD) were constructed. The results showed that esterification modification reduced the relative crystallinity and short-range order of starch but significantly increased the content of resistant starch (RS) and thermal processing stability, indicating that the ordered regions of starch molecules were reconstructed to form a structural system with better anti-digestion properties. With the increase in the degree of esterification substitution, the surface of starch granules gradually became rough, the particle size increased, the solubility and swelling power decreased, and the contact angle increased. EGSC30 could synergistically stabilize Pickering HIPEs with β-CD. As the total concentration of composite particles increased and the β-CD/EGSC30 mass ratio was optimized to 3:1, the droplet size of Pickering emulsions decreased, the gel strength and storage modulus increased significantly, and the system exhibited typical elastic-dominant gel properties and shear-thinning behavior, with the most compact and stable network structure. The obtained results can promote the deep processing of ginger and provide a reference for the construction of novel food-grade Pickering emulsions.

## 1. Introduction

Starch is a widely available, renewable, and low-cost natural polymeric polysaccharide that is extensively used in the food industry for thickening, gel formation, emulsion stabilization, and texture modification. Native starch granules typically exhibit poor solubility, limited water retention, insufficient emulsifying stability, and a tendency to retrograde during processing. Therefore, structural and functional modifications via chemical, enzymatic, or physical methods are required to meet specific food processing needs. Negi et al. utilized α-amylase and glycosyl transferase to alter the molecular weight distribution, amylose content, and crystallinity of starch, thereby modulating its digestibility, rheological properties, and gelation behavior [1]. Lin et al. summarized the physical techniques that can significantly enhance starch functionality—such as water-holding capacity, freeze–thaw stability, and anti-retrogradation—by modifying molecular arrangement and granule structure [2].

Citric acid is a food-grade natural polycarboxylic acid that is safe, environmentally friendly, and low-cost, widely used in food processing and polysaccharide modification. Its esterification reaction is mild and solvent-free, making it an eco-friendly method for starch modification [3]. Cavallo et al. introduced citric acid groups to modulate the hydrophilic–lipophilic balance and thermodynamic properties of starch, thereby improving its mechanical, pasting, and emulsifying properties [4]. Zheng et al. found that citric acid modification reduced starch hydrophilicity, decreased crystallinity, and increased resistant starch content, providing a theoretical basis for developing low-digestibility and functional foods [5]. Lee et al. reported that citric acid esterification could enhance starch granule swelling and interfacial adsorption capacity, thus improving emulsion stability and textural characteristics [6].

Pickering high internal phase emulsions (HIPEs), stabilized by solid particles instead of traditional surfactants, have attracted widespread attention in food and functional formulations due to their excellent biocompatibility and stability. Natural biopolymer particles such as proteins, polyphenols, and polysaccharides are commonly used to stabilize Pickering HIPEs. However, native polysaccharide particles are typically highly hydrophilic, making it difficult to form a robust adsorption layer at the oil–water interface, thus limiting emulsion stability. Citric acid esterification partially replaces hydrophilic hydroxyl groups on starch molecules with citrate ester moieties, thereby reducing surface polarity and significantly enhancing particle interfacial activity and emulsion stability, while maintaining the natural safety and edibility of starch [7]. Chen et al. further demonstrated that citric acid-modified rice starches from different sources effectively improved the interfacial structure of emulsions, thereby enhancing the overall stability of Pickering emulsions [8]. Cyclodextrins, known for their unique cyclic structure and hydrophobic cavity, are valuable in food structure modulation and functional molecule encapsulation. Among them, β-cyclodextrin, with good biocompatibility, is often used as an emulsion stabilizer. However, previous studies have mainly focused on citric acid-modified starches as individual Pickering stabilizers or β-CD-based composite systems with other polysaccharides [8,9]. How citric acid-esterified starch with tailored wettability cooperates with β-CD to stabilize Pickering HIPEs and form emulsion gel networks remains insufficiently understood.

Ginger starch (GS) is an underutilized botanical starch resource with distinctive oval granules and relatively small particle size. These features may favor interfacial adsorption and particle network formation. However, its weak processing stability limits practical applications, and little is known about how citric acid esterification regulates the structure, wettability, and Pickering emulsifying capacity of GS. In this study, citric acid-esterified ginger starch (EGSC) was prepared via a dry-heat method, and its morphological, structural, and functional changes were systematically characterized. A Pickering high internal phase emulsion (HIPE) was fabricated using β-CD/EGSC hybrid particles, and the effects of particle composition and dosage on emulsion gel properties were investigated. These findings provide new insight into the structure–wettability–interfacial stabilization relationship of citric acid-esterified GS and offer a feasible strategy for constructing starch-based composite particles for food-grade Pickering HIPEs.

## 2. Materials and Methods

### 2.1. Materials and Chemicals

Ginger starch (GS) was prepared following the protocol described in our earlier work [10]. Citric acid, β-cyclodextrin, and medium-chain triglyceride (MCT) were obtained from Shanghai Yuanye Bio-Technology Co., Ltd. (Shanghai, China), while sunflower oil was sourced from COFCO Fortune Co., Ltd. (Beijing, China). Amyloglucosidase (AMG) and α-amylase were supplied by Yisijiu Bio-Technology Co., Ltd. (Lianyungang, China).

### 2.2. Preparation of EGSC

Ginger starch (GS) was prepared according to our previous method. Briefly, 500 g of fresh ginger was peeled, cut into small pieces, and homogenized using a high-speed blender. The resulting slurry was filtered through a 200-mesh gauze bag to remove fibrous residues. The filtrate was then allowed to stand overnight for natural sedimentation, and the lower white precipitate was collected as crude ginger starch. The precipitate was dried in an oven at 40 °C to constant weight, ground, and passed through a 200-mesh sieve to obtain GS.

EGSC was prepared using the dry-heat method [8]. Specifically, 2 g, 4 g, or 6 g of citric acid was dissolved separately in 15 mL of distilled water, and the pH of each solution was adjusted to 3.0 using NaOH before bringing the final volume to 20 mL. Each prepared solution was then thoroughly mixed with 20 g of GS and allowed to equilibrate at 30 °C for 16 h. The resulting mixtures were oven-dried at 45 °C for 24 h, ground, and further heated at 130 °C for 5 h. The products were subsequently washed three times with distilled water and once with absolute ethanol, followed by drying at 45 °C, grinding, and passing through a 200 mesh sieve. The final citric acid-esterified GS samples were designated EGSC10, EGSC20, and EGSC30, corresponding to the three citric acid dosages employed.

### 2.3. Determination of Substitution Degree

The substitution degree of EGSC was determined by the titration method [11]. In brief, 1 g of EGSC was suspended in 10 mL of distilled water, to which three drops of 1% phenolphthalein were added as an indicator. The suspension was initially titrated with 0.1 M NaOH until a faint pink endpoint remained. Thereafter, 5 mL of 0.5 M NaOH was introduced, and the mixture was stirred magnetically for 1 h. Following this, the solution was back-titrated with 0.5 M HCl until the pink color disappeared. A parallel blank determination was carried out under identical conditions using unmodified GS. The degree of substitution was subsequently calculated according to the equations provided below:

(1) $$A\%\;=\;\frac{{(V}_{0}-V_{1})\times M\times 0.158\times 100}{W}$$

(2) $$DS=\frac{162\times A}{15800-156\times A}$$

In this equation, *A* represents the mass fraction (%) of the citric acid substituent, *M* denotes the molarity of the HCl solution, *W* corresponds to the starch mass (g), *V*_1_ is the volume of HCl solution used to titrate the EGSC sample, and *V*_0_ refers to the volume required for titrating the blank. The constants 0.158, 162, 15,800, and 156 correspond to the mass conversion factor of the citric acid substituent, the molecular weight of the anhydroglucose unit, the percentage conversion term, and the net molecular weight contribution of the citric acid substituent after esterification, respectively.

### 2.4. Determination of Particle Morphology

The surface topography of GS and EGSC particles was examined using a ZEISS Sigma 300 scanning electron microscope (Jena, Germany). A suitable quantity of starch particles was uniformly spread onto conductive adhesive tape and mounted onto the specimen holder. To improve electrical conductivity, the sample was sputter-coated with gold under vacuum conditions. Afterwards, micrographs were acquired at an acceleration voltage of 3.0 kV and a magnification of 1000×, allowing the surface structural features of the starch particles to be assessed [12].

### 2.5. Determination of Particle Size Distribution

The particle size distribution of GS and EGSC was evaluated using a BT-9300H laser particle size analyzer (Bettersize, Dandong, China). Each starch sample was added to the instrument’s stirrer and thoroughly dispersed in distilled water, with the suspension transmittance adjusted to 15–18%. Measurements were conducted continuously under stable conditions, and the resulting data were used to characterize the particle size distribution of the samples [13].

### 2.6. X-Ray Diffraction Measurement

The X-ray diffraction (XRD) patterns of GS and EGSC were obtained using a Bruker D8 Advance diffractometer (Billerica, MA, USA) employing a step-scan technique. Measurements were performed over a 2θ range of 5–80° with a step size of 0.02°, using a Cu target operated at 40 kV and 100 mA, and a scanning rate of 8°/min. The relative crystallinity of the samples was subsequently determined using Jade6 software [14].

### 2.7. Fourier Transform Infrared (FT-IR) Spectroscopy Measurement

For Fourier transform infrared (FT-IR) measurements, each starch sample was thoroughly mixed with KBr and pressed into pellets. Spectra were recorded on a Thermo Scientific Nicolet iS20 spectrometer (Waltham, MA, USA) across the 500–4000 cm^−1^ range, using a resolution of 4 cm^−1^ and accumulating 64 scans per sample. After applying baseline correction and spectral deconvolution, the absorbance intensities at 1022 cm^−1^ and 1047 cm^−1^ were extracted, and the ratio of R_1047/1022_ was calculated to assess structural features [15].

### 2.8. Determination of Solubility and Swelling Power

An appropriate amount of starch sample (W_initial_) was dispersed in water to form a 2% (*w*/*w*) starch suspension, heated in a shaking water bath at 80 °C for 30 min, and then rapidly cooled to room temperature. The suspension was subjected to centrifugation at 4000 r/min for 15 min. The resulting supernatant was collected in a pre-weighed glass dish and dried at 105 °C to a constant weight to determine the mass of soluble starch (W_soluble_). The mass of the pellet obtained after centrifugation was taken as the swollen starch mass (W_swollen_) [16]. Solubility (SOL) and swelling power (SP) were subsequently calculated according to the following equations:

(3) $$\mathrm{SOL}\;=\;\frac{W_{\mathrm{soluble}}}{W_{\mathrm{initial}}}\times 100\%$$

(4) $$\mathrm{SP}\;=\frac{W_{\mathrm{swollen}}}{W_{\mathrm{initial}}(1-\mathrm{SOL})}\times 100\%$$

### 2.9. Determination of Resistant Starch Content

The resistant starch (RS) contents of GS and EGSC were determined according to the method of Liu et al. [17]. The resistant starch (RS) content was quantified using a sequential enzymatic approach. Each starch sample (100 mg) was initially incubated with α-amylase and amyloglucosidase (AMG) in a shaking water bath at 37 °C for 16 h. The enzymatic reaction was then terminated by adding an equal volume of ethanol. Following centrifugation, the supernatant was discarded, and the remaining insoluble flocculent was collected as the RS fraction. This residue was washed twice with 50% (*v*/*v*) ethanol, with centrifugation and removal of the supernatant performed after each wash. Subsequently, 2 mL of 2 M KOH was added to fully solubilize the pellet, and the solution was neutralized by the addition of an acetate buffer. The dissolved starch was then converted quantitatively to glucose by incubating with AMG at 50 °C for 30 min, yielding the RS solution. A 0.1 mL aliquot of this RS solution was mixed with 3 mL of the glucose oxidase/peroxidase (GOPOD) reagent, vortexed, and incubated at 50 °C for 20 min. A glucose standard solution (1 mg/mL, 0.1 mL) served as the positive control, while 0.1 mL of the 0.1 M sodium acetate buffer combined with 3 mL of the GOPOD reagent was used as the blank. Absorbance readings were taken at 510 nm using a colorimetric method, and the RS content was calculated based on these measurements.

For the “After cooking” procedure, 100 mg of the starch sample was combined with 4 mL of distilled water and heated in a boiling water bath for 30 min. Upon completion of cooking, the mixture was cooled to below 37 °C, after which the subsequent steps were carried out following the “Before cooking” protocol. These steps included enzymatic digestion, centrifugation, washing, dissolution, hydrolysis, and glucose quantification, enabling the determination of resistant starch (RS) content after cooking.

### 2.10. Contact Angle Measurement

The three-phase contact angle of GS and EGSC was determined using a Theta Lite optical contact angle goniometer (Biolin Scientific AB, Göteborg, Sweden). Prior to measurement, the samples were compressed into thin films under a pressure of 15 MPa using a tablet press. The resulting films were then placed in quartz cuvettes filled with medium-chain triglyceride (MCT). A 10 μL droplet of ultrapure water was gently deposited onto the film surface using a high-precision microsyringe (Biolin Scientific AB, Göteborg, Sweden). The morphology of the droplet was continuously monitored and recorded for 10 s at a rate of 1 frame per second. During this process, the instrument automatically captured the droplet images and calculated the corresponding contact angle values.

### 2.11. Preparation of Pickering Emulsions

To formulate the Pickering emulsions, aqueous phases were prepared using different total particle concentrations and β-CD/EGSC30 mass ratios, with sunflower oil acting as the oil phase. Here, the total particle concentration refers to the combined mass concentration of β-CD and EGSC30 in the aqueous phase, expressed as the total mass of β-CD and EGSC30 per unit volume of the aqueous phase. The two immiscible components were homogenized at ambient temperature using an IKA Ultra-Turrax T18 high-speed homogenizer (Staufen, Germany) operated at 15,000 rpm for 3 min. Upon standing, a series of Pickering emulsions was generated by varying three parameters: the oil volume fraction (φ = 60%, 65%, 70%, 75%, and 80% *v*/*v*), the total particle concentration (c = 0.5%, 1.0%, and 1.5% *w*/*v*), and the β-CD/EGSC30 mass ratio (k = 4:0, 3:1, 2:2, 1:3, and 0:4 *w*/*w*). After storage at room temperature for 24 h, the preliminary short-term stability of the resulting emulsions was assessed by visual observation, inversion behavior, and phase separation [9].

### 2.12. Measurement of Droplet Size of Pickering HIPEs

The oil droplet size of the emulsion was measured using a laser diffraction particle size analyzer at 25 °C. Before measurement, the emulsion sample was appropriately diluted with deionized water and introduced into the measurement cell under continuous stirring. Measurements were performed when the sample obscuration reached 15–16%, ensuring suitable light scattering intensity for droplet size analysis. The droplet size distribution was automatically calculated by the instrument based on the diffraction pattern of the dispersed oil droplets.

### 2.13. Observation of Emulsion Morphology

An aliquot of emulsion (20 μL) was deposited onto a glass microscope slide and gently compressed with a coverslip to form a thin layer. The microstructure of the emulsion droplets was then examined using a BH200P optical microscope equipped with a digital imaging system (Sunny Optical Instrument Co., Ltd., Ningbo, China).

### 2.14. Measurement of Gel Strength

The gel strength of the Pickering HIPEs was determined using a TA-XT Plus texture analyzer (Stable Micro Systems, Godalming, UK) operating in GMIA Gelation mode and fitted with a P/0.5 probe with a diameter of 12.7 mm. During the measurement, the probe descended at a speed of 1.0 mm/s until a trigger force of 3.0 g was reached, followed by a further downward displacement of 4 mm. The resulting data were automatically collected and subsequently analyzed using Exponent 6.1 software [18].

### 2.15. Measurement of Rheological Properties

The rheological properties of the Pickering high internal phase emulsions (HIPEs) were evaluated using a HAAKE MARS III rotational rheometer equipped with a 35 mm P35TiL parallel plate geometry (Thermo Fisher Scientific, Waltham, MA, USA). A measuring gap of 1 mm was maintained between the rotor and the sample stage, and all measurements were conducted at 25 °C. Oscillatory stress sweeps were first performed at a fixed frequency of 1 Hz across a shear stress range of 1–100 Pa. Using the linear viscoelastic region identified from strain sweep experiments, frequency ramp tests were subsequently carried out over 1–100 Hz to determine the storage modulus (G′) and loss modulus (G″). Furthermore, to examine how the apparent viscosity varied with shear rate, shear rate ramp measurements were performed under a constant stress of 5 Pa over a shear rate interval of 1–10 s^−1^ [19].

### 2.16. Statistical Analysis

All measurements were conducted in triplicate, and the results are presented as mean values accompanied by their corresponding standard deviations. Statistical analyses were carried out using SPSS 20.0 software. Before one-way ANOVA, the normality of the data and homogeneity of variances were checked. Differences among groups were evaluated by one-way analysis of variance (ANOVA), followed by Duncan’s multiple range test for post hoc comparisons. A probability level of *p* < 0.05 was considered indicative of statistical significance. Graphical representations of the data were prepared using Origin 2021 software.

## 3. Results and Discussion

### 3.1. Substitution Degree Analysis

The degree of substitution (DS) of esterified starch is a key indicator for evaluating the extent of starch esterification and significantly influences its molecular structure and physicochemical properties [11]. This study investigated the effect of citric acid dosage (10%, 20%, and 30%) on the DS of EGSC (Table 1). As the citric acid dosage increased, the DS of EGSC gradually rose, with DS values of 0.060 ± 0.01, 0.088 ± 0.01, and 0.110 ± 0.01 for EGSC10, EGSC20, and EGSC30, respectively, and significant differences were observed among samples (*p* < 0.05). These results indicate that increasing the citric acid dosage effectively promotes the esterification reaction. A higher citric acid concentration increases the number of carboxyl groups available for reaction, thereby enhancing the probability of esterification with hydroxyl groups on starch molecules and leading to more hydroxyls being replaced by citric acid to form ester linkages. Remya et al. also found that citric acid esterification could form ester bonds or cross-linked structures between starch chains [20].

### 3.2. Analysis of Particle Morphology

**Figure 1:**
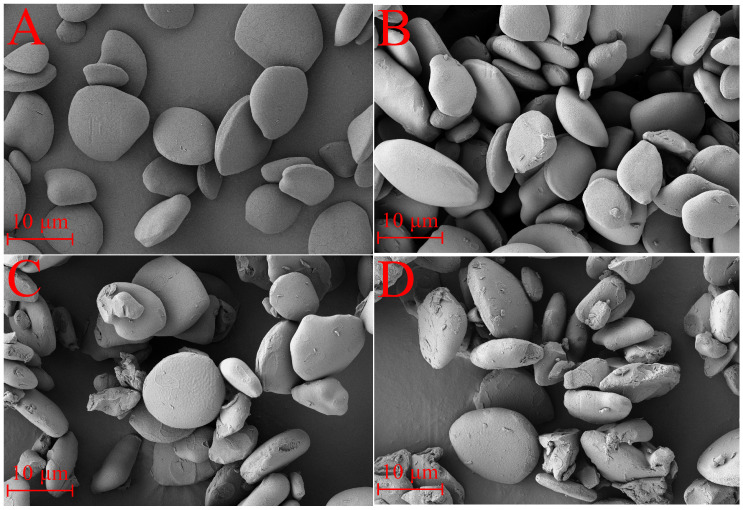
The SEM images of GS (**A**) and EGSC10 (**B**), EGSC20 (**C**), and EGSC30 (**D**).

SEM images are shown in Figure 1. GS particles exhibit regular elliptical or oval shapes with smooth surfaces and clear boundaries, without obvious cracks or structural collapse. After citric acid esterification, EGSC10, EGSC20, and EGSC30 largely retain their original morphology, with no severe fragmentation or complete collapse observed, indicating that the esterification conditions used in this study fall within a controllable modification range. However, compared to GS, the surface of EGSC particles gradually becomes rougher, with slight indentations and irregular structures appearing at the edges of some particles. This surface heterogeneity becomes more pronounced as the degree of substitution increases. In EGSC10, slight etching marks begin to appear on the particle surface, though the overall structure remains relatively intact. In EGSC20, surface roughness further increased, and localized structural loosening was observable. For EGSC30, the particles displayed more pronounced surface irregularities and mild deformation at some edges, yet no complete rupture or collapse occurred. These observations suggest that, under dry-heat conditions, esterification mainly occurred at accessible hydroxyl groups on the granule surface and in amorphous regions, rather than uniformly throughout the whole granule. The ester bonds formed between citric acid and starch chains may partially disrupt the intermolecular hydrogen-bonding network and reduce the compactness of the outer granule structure, which manifests as increased surface roughness and localized structural loosening [21].

### 3.3. Analysis of Particle Size Distribution

As shown in Table 1, the particle size of GS is 10.90 ± 0.09 μm. After citric acid esterification, a significant increase in particle size is observed (*p* < 0.05), with EGSC10, EGSC20, and EGSC30 reaching 12.86 ± 0.14 μm, 14.21 ± 0.09 μm, and 20.48 ± 0.11 μm, respectively. As the degree of substitution increases from 0.060 to 0.110, the particle size rises in a stepwise manner, with a particularly notable increase in EGSC30. This indicates that esterification not only alters the internal molecular structure of starch but also affects the external state of the particles. Combined with SEM results, although the overall morphology of esterified particles remains intact, increased surface roughness and localized structural loosening are evident. These surface changes may enhance interparticle interactions, potentially leading to particle aggregation and thus an increase in apparent particle size within the dispersion system [22].

### 3.4. XRD Analysis

**Figure 2:**
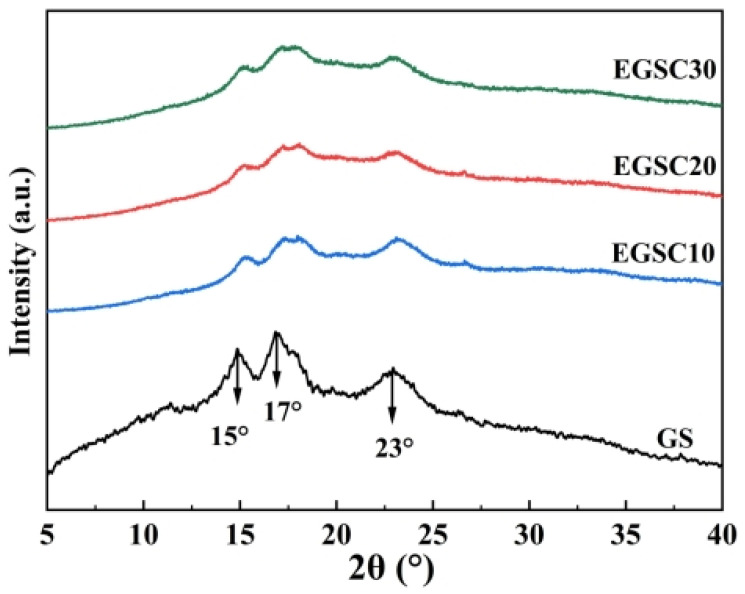
XRD patterns of GS, EGSC10, EGSC20, and EGSC30.

X-ray diffraction (XRD) analysis was conducted to investigate the crystalline architecture of the samples, providing insight into how esterification influenced the ordered arrangement and crystalline characteristics of the starch regions [4]. The XRD patterns of GS, EGSC10, EGSC20, and EGSC30 are shown in Figure 2. GS exhibits characteristic diffraction peaks at approximately 2θ = 15°, 17°, and 23°, indicating a typical A-type crystalline structure. After citric acid esterification, the main diffraction peak positions of all modified samples remain largely unchanged, suggesting that the esterification reaction does not alter the crystal type of GS. However, after citric acid esterification, the characteristic diffraction peaks became less pronounced, and the relative crystallinity decreased progressively with increasing degree of substitution. Meanwhile, the relative crystallinity decreases from 24.83% in GS to 17.3%, 14.7%, and 12.5% in EGSC10, EGSC20, and EGSC30, respectively. This indicates that the introduction of citric acid groups disrupts the ordered molecular arrangement within the starch crystalline regions, thereby reducing the structural order of the crystals. This may be attributed to the formation of ester bonds altering the inter- and intramolecular hydrogen bonding networks in starch, leading to a less dense crystalline layer structure. Additionally, the relatively large volume of citric acid groups introduces steric hindrance, which restricts the tight packing of amylopectin double helices, resulting in a gradual loosening of the crystalline structure and ultimately a reduction in crystallinity. This finding is consistent with the results reported by Lv et al. [11], who demonstrated that the penetration and chemical reaction of citric acid during esterification disrupt the ordered structure of starch molecules, leading to a significant reduction in ordered crystalline domains.

### 3.5. Structural Characteristics

**Figure 3:**
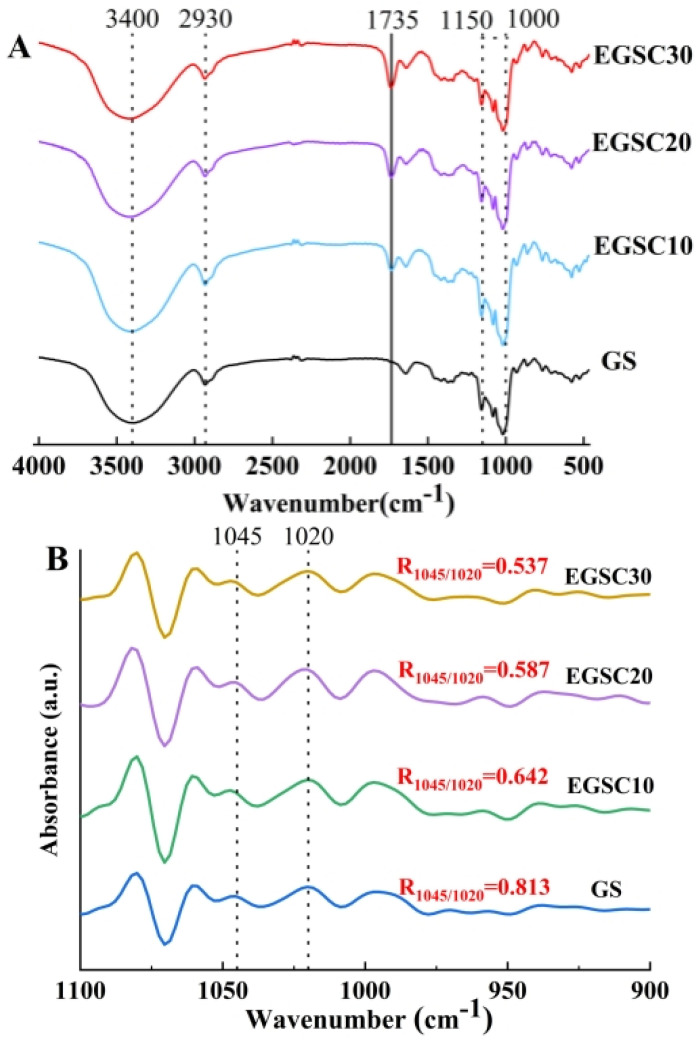
FT-IR (**A**) and infrared deconvolution spectra (**B**) of GS, EGSC10, EGSC20, and EGSC30.

The FT-IR spectra of GS and EGSC are depicted in Figure 3A. In the case of GS, a broad and pronounced absorption band appeared around 3400 cm^−1^, corresponding to the stretching vibrations of hydroxyl (–OH) groups within the starch molecules. The peak observed near 2930 cm^−1^ was attributed to C–H stretching vibrations. Moreover, several characteristic bands in the 1150–1000 cm^−1^ range were associated with C–O–C and C–O stretching vibrations, representing the intrinsic structural features of starch. In contrast, EGSC displayed an additional absorption band at approximately 1735 cm^−1^, the intensity of which increased with higher citric acid concentrations. This band is indicative of the C=O stretching vibration of ester groups, providing clear evidence of esterification between citric acid and the starch, thereby confirming the successful formation of ester linkages [8]. This observation aligns with the substitution degree results, demonstrating that the extent of esterification increases with higher citric acid dosage.

To further evaluate the impact of citric acid esterification on the short-range ordered structure of starch, deconvolution of the IR spectrum in the 1100–900 cm^−1^ region was performed (Figure 3B). This region is closely related to the double-helical conformation and local ordered structure of starch, where the 1045 cm^−1^ peak reflects the ordered crystalline domains and the 1022 cm^−1^ peak primarily corresponds to amorphous regions. The R_1045/1022_ value for GS is 0.813, indicating a high degree of short-range molecular order. After esterification, this ratio significantly decreases to 0.642 (EGSC10), 0.587 (EGSC20), and 0.537 (EGSC30), showing a continuous decline with increasing degree of substitution. The progressive reduction in R_1045/1022_ indicates that esterification weakens the local ordered arrangement of starch molecules and reduces the stability of double helices. This change is likely due to the disruption of intermolecular hydrogen bonding networks and steric hindrance caused by the introduced citric acid groups, which restrict tight packing and reorganization of starch chains. Notably, the decreasing trend in short-range order correlates well with the XRD results showing a decline in crystallinity from 24.83% to 12.5%, indicating that citric acid esterification affects both the short-range and long-range structural order of starch.

### 3.6. Analysis of Solubility and Swelling Power

The solubility and swelling power of GS and EGSC were measured to evaluate the impact of esterification on starch–water interactions and the structural stability of starch granules. The solubility and swelling power of GS were 8.97 ± 0.82% and 6.86 ± 1.11%, respectively (Table 1). After citric acid esterification, both solubility and swelling power significantly decreased (*p* < 0.05). For EGSC10, EGSC20, and EGSC30, solubility dropped to 5.82 ± 0.44%, 2.76 ± 0.25%, and 1.38 ± 0.17%, while swelling power decreased to 4.90 ± 0.10%, 3.74 ± 0.17%, and 2.97 ± 0.06%, respectively. With increasing degree of substitution, both parameters showed a continuous downward trend, indicating that citric acid esterification markedly suppresses the water uptake and swelling capacity of starch granules. This change is primarily attributed to the formation of ester bonds introducing cross-linked structures between starch molecules, which restricts chain mobility and enhances the internal structural stability of the granules. Combined with FTIR and XRD results, although esterification reduces the ordered structure of starch, the citric acid molecules act as bridges between starch chains via their multi-carboxyl structure, leading to a denser granule architecture that hinders water penetration and thereby reduces swelling behavior [3].

### 3.7. RS Content Analysis

Measuring the resistant starch (RS) content of GS and EGSC helps evaluate the impact of esterification on starch digestibility and structural stability. As shown in Table 2, the RS content of GS is 34.05%, which significantly decreases to 15.53% after cooking, indicating that starch undergoes complete gelatinization during heating, disrupting its original ordered crystalline structure and thereby increasing susceptibility to enzymatic hydrolysis. Esterification markedly affected RS content in a dosage-dependent manner. Before cooking, RS content gradually rises from 30.64% (EGSC10) to 48.14% (EGSC30), indicating that higher degrees of esterification favor the formation of enzyme-resistant structures. The lower RS content of EGSC10 compared with native GS may be due to the limited esterification degree, in which mild acid/thermal treatment partially disrupted the native ordered regions but did not provide sufficient ester bond-mediated structural constraints to resist enzymatic hydrolysis. At higher citric acid dosages, the increase in RS content was primarily attributed to ester bond-mediated cross-linking and the introduction of citrate ester moieties, which restrict starch chain mobility and enhance the rigidity of the internal granule structure.

After cooking, RS content decreases in all samples, but EGSC remains significantly higher than GS. Notably, EGSC30 retains a high level of 44.62% post-heating, demonstrating excellent thermal stability. This suggests that esterification effectively enhances the starch structure’s resistance under thermal processing conditions. The effect may be due to citric acid-induced structural constraints that limit granule swelling and gelatinization, thereby reducing enzyme accessibility to starch molecules. This finding aligns with the observed decline in short-range molecular order in FT-IR analysis and the reduced solubility and swelling power, indicating that esterification promotes structural reorganization into a denser, digestion-resistant architecture [5].

### 3.8. Three-Phase Contact Angle Analysis

**Figure 4:**
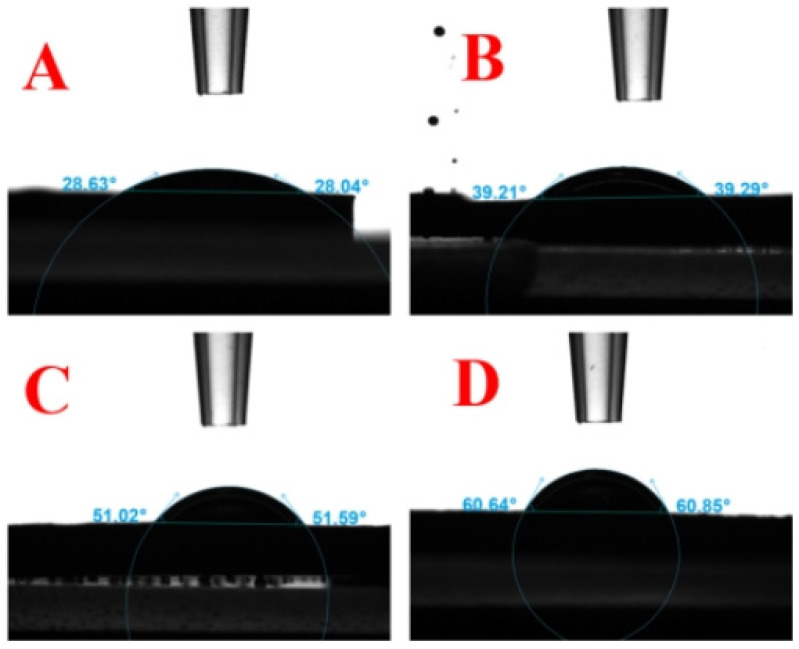
Three-phase contact angles of GS (**A**), EGSC10 (**B**), EGSC20 (**C**) EGSC30 (**D**).

The images in Figure 4 show the three-phase contact angles of GS, EGSC10, EGSC20, and EGSC30 in the MCT system. The contact angle of GS is approximately 28.6°, indicating strong hydrophilicity of its surface. After citric acid esterification, the contact angles of EGSC10, EGSC20, and EGSC30 increase to about 39.3°, 51.3°, and 60.7°, respectively, showing a rising trend with increasing degree of substitution. The increased contact angle indicates that esterification introduces ester groups onto starch molecules, partially replacing hydroxyl groups and reducing surface polarity, thereby enhancing the hydrophobicity of the particles. Studies have shown that solid particles with contact angles between 30° and 90° at the oil–water interface are more favorable for stabilizing Pickering emulsions [23]. Within this contact angle range, particles generally exhibit a more appropriate oil–water wettability balance. Unlike highly hydrophilic particles that tend to remain in the aqueous phase, they can be partially wetted by both oil and water and anchor more firmly at the three-phase contact line, thereby increasing desorption energy and improving Pickering emulsion stability. EGSC30 exhibits a higher contact angle and more suitable interfacial wettability, suggesting stronger adsorption capacity at the oil–water interface. Therefore, EGSC30 was selected as the stabilizing particle for constructing the subsequent Pickering emulsion system.

### 3.9. Formation of Pickering Emulsions

**Figure 5:**
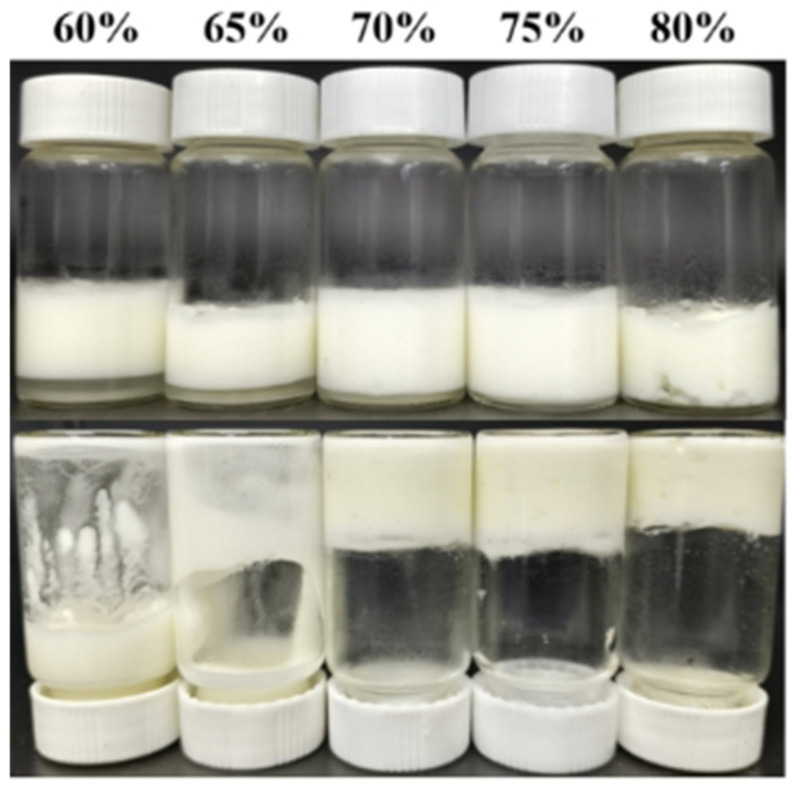
Effect of the oil phase volume fraction on formation of Pickering emulsion gels.

Identifying the optimal values for oil phase volume fraction (*φ*), total particle concentration (*c*), and β-CD/EGSC30 mass ratio (*k*) is essential for the successful preparation of Pickering emulsions. Among these parameters, φ plays a particularly critical role, as it directly affects the emulsion type, stability, droplet dimensions, and microstructural characteristics [24]. Figure 5 shows Pickering emulsions formed by β-CD/EGSC30 mixtures at *c* = 1.0% and *k* = 3:1 under different oil phase volume fractions (*φ* = 60%, 65%, 70%, 75%, 80%). At φ = 60% and 65%, the emulsions flowed upon inversion, as the internal phase fraction was too low to form a continuous particle network. At φ = 80%, insufficient particle coverage at the oil–water interface caused surface oil separation. In contrast, at φ = 70% and 75%, particles effectively covered droplet surfaces and formed a continuous 3D network, resulting in stable gel-like emulsions. According to the general definition of high internal phase emulsions (HIPEs, φ ≥ 74%), only the φ = 75% system qualified as a Pickering HIPE and was used for subsequent experiments [25]. These emulsions exhibit solid-like behavior, high oil-loading capacity, and tunable rheological properties, enabling greater encapsulation of active ingredients or nutrients and texture modulation through viscoelastic control [26]. The observation that a stable Pickering emulsion could be formed at *φ* = 75% confirms that the β-CD/EGSC30 combination exhibits direct emulsifying capacity for constructing Pickering HIPEs. Consequently, this specific oil volume fraction of 75% was adopted as the fixed parameter for the formulation of HIPEs.

**Figure 6:**
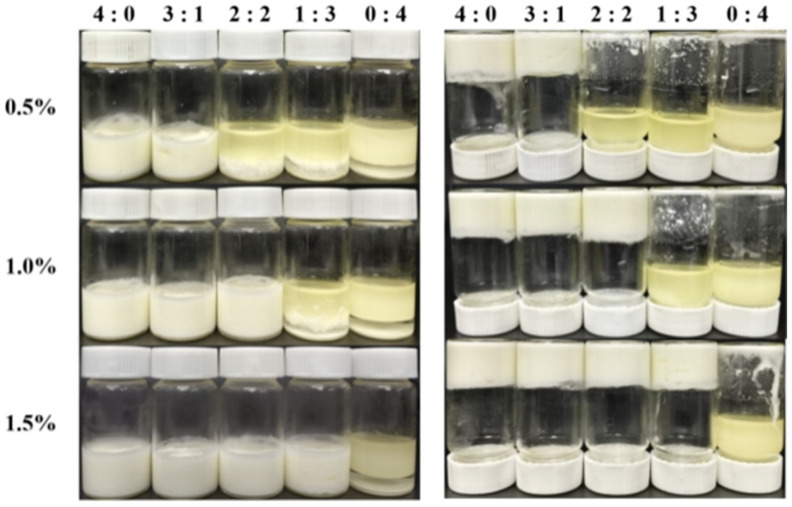
Effect of the composition and concentration of β-CD/EGSC30 mixture on formation of Pickering HIPEs.

Figure 6 presents the effects of the mixture concentration (*c*) and the mass ratio (*k*) of β-CD/EGSC30 on the formation of Pickering HIPEs at an oil phase volume fraction of 75%. The results indicate that when the mixture concentration was maintained at 0.5%, stable HIPE systems could only be obtained at mass ratios of *k* = 4:0 and 3:1. When *c* increases to 1.0%, the stable range expands to include *k* = 4:0, 3:1, and 2:2. At *c* = 1.5%, stable HIPEs are formed across *k* = 4:0, 3:1, 2:2, and 1:3, indicating that higher particle concentration promotes structural stability. Notably, no stable emulsion forms at *k* = 0:4 under any concentration, suggesting that EGSC30 alone cannot effectively stabilize the HIPEs. In contrast, the addition of EGSC30 to β-CD does not impair emulsification, implying a synergistic interfacial stabilization effect. This may arise from the preferential adsorption of β-CD at the oil–water interface and the reinforcement of the interfacial film by EGSC30, collectively enhancing system stability. Based on these findings, representative samples were selected for further analysis of microstructure and mechanical properties to elucidate the synergistic stabilization mechanism of β-CD and EGSC30 in Pickering HIPEs. It should be noted that this 24 h assessment only reflects the preliminary short-term physical stability of the Pickering HIPEs, whereas their long-term storage stability requires further investigation.

### 3.10. Droplet Size and Optical Microscopy Analysis

**Figure 7:**
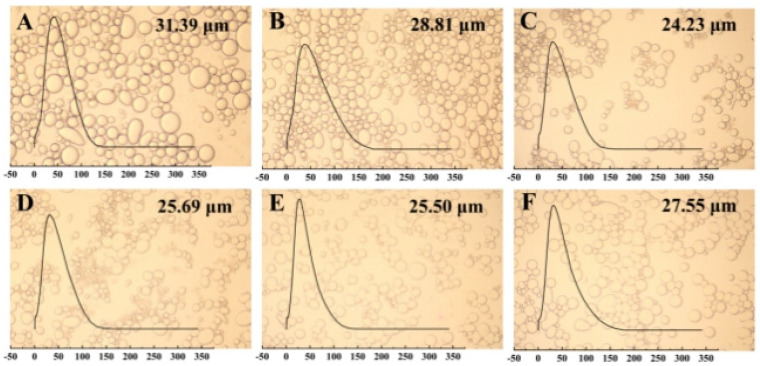
Effect of the composition and concentration of β-CD/EGSC30 mixture on droplet appearance of HIPEs (**A**, **B** and **C** are the emulsions developed at φ = 75%, k = 3:1, c = 0.5%, 1.0% and 1.5%, respectively; **D**, **E** and **F** are the emulsions developed at φ = 75%, c = 1.5%; k = 4:0, and 2:2 and 1:3, respectively).

Emulsion droplet size is a key indicator for evaluating physical stability and uniformity of droplet distribution, directly influenced by particle emulsification efficiency and interfacial film strength. Figure 7 shows the effect of concentration (c) and mass ratio (k) on the droplet size and microstructure of HIPEs [27]. At a mass ratio of k = 3:1, increasing the particle concentration from 0.5% to 1.5% led to a pronounced reduction in droplet diameter, decreasing from 31.39 μm to 24.23 μm. This effect can be attributed to the greater availability of particles capable of adsorbing at the oil–water interface, which enhances interfacial coverage, lowers interfacial tension, and consequently inhibits droplet coalescence during the emulsification process. When the particle concentration reached 1.5%, the droplet sizes obtained at k = 4:0, 3:1, and 2:2 were all relatively small (approximately 25 μm), and no significant differences were observed among these systems, indicating that they exhibited comparable and efficient emulsifying capacity. However, when the ratio shifts further toward starch (k = 1:3), droplet size significantly increases to 28.46 μm. This trend suggests that β-CD plays a dominant role in droplet refinement, while excessive starch may increase system viscosity or reduce coverage efficiency due to larger particle size and slower adsorption kinetics, leading to partial droplet aggregation [28].

### 3.11. Gel Strength Analysis

**Figure 8:**
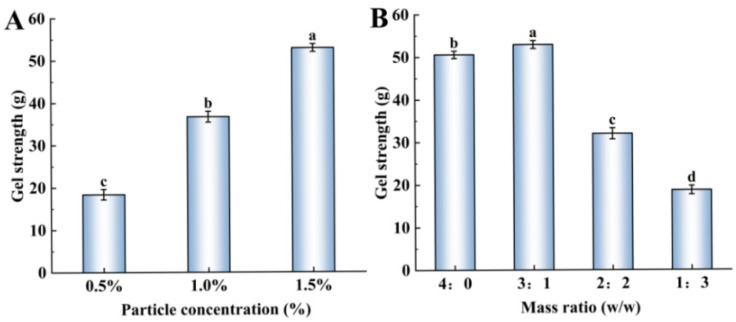
Effect of β-CD/EGSC30 mixture concentration (**A**) and composition (**B**) on the gel strength of Pickering HIPEs. (Different lowercase letters above the bars indicate significant differences (*p* < 0.05)).

Gel strength, as a core physical indicator for evaluating the stability of the emulsion gel’s 3D network framework and its mechanical processability, directly reflects the degree of intermolecular interaction between EGSC30 and β-CD. Figure 8A shows that as the total particle concentration increases from 0.5% to 1.5%, the gel strength of the emulsion gradually increases. This is primarily because, at higher concentrations, solid particles more fully occupy the oil–water interface and continuous phase, enhancing particle–particle contact and connectivity, thereby forming a denser network structure. From the results at different k values (Figure 8B), the gel strength reaches its maximum at *k* = 3:1, showing a clear synergistic enhancement trend. This indicates that an appropriate amount of EGSC30 improves the spatial arrangement of composite particles within the system, leading to a more compact network structure [19]. However, when the proportion of EGSC30 further increases (*k* = 2:2, 1:3), the gel strength decreases significantly. It is speculated that excess EGSC30 disrupts the original particle packing state of β-CD, and under the conditions of this study, the structural strength formed by EGSC30 alone is limited and insufficient to support a high-strength emulsion framework, resulting in weakened overall mechanical performance.

### 3.12. Analysis of Rheological Properties

**Figure 9:**
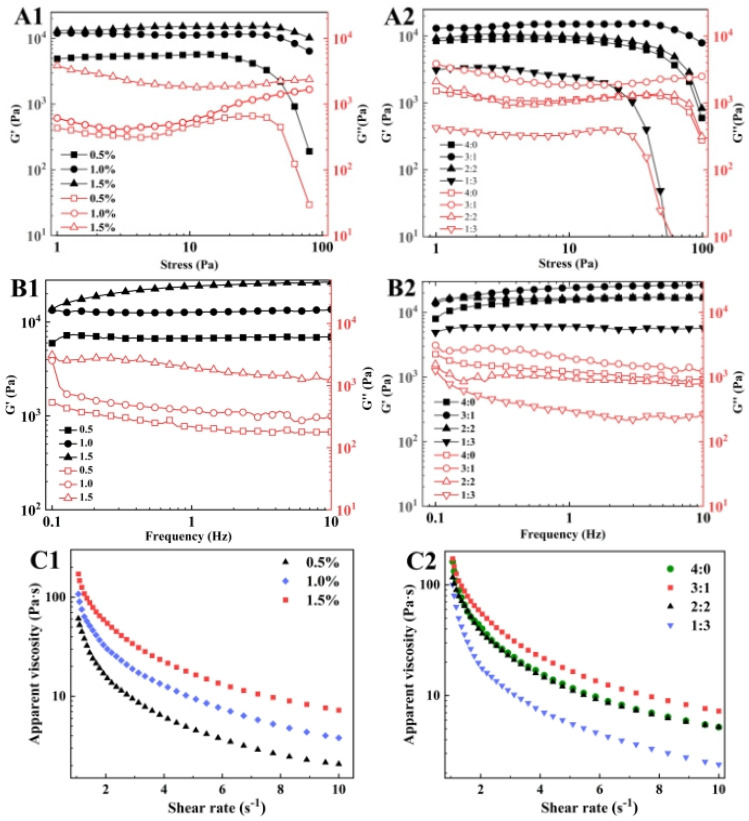
Effect of β-CD/EGSC30 mixture concentration and composition on the rheological properties of Pickering HIPEs (**A1**,**A2**,**B1**,**B2**,**C1**,**C2**).

The rheological properties of emulsion gels reflect their macroscopic flow and deformation behavior and are closely related to the system’s microstructure, serving as a key indicator for evaluating the structural stability and network strength of Pickering HIPEs. The storage modulus (G′) characterizes the elastic component of a material, whereas the loss modulus (G″) describes its viscous behavior. When the value of G′ exceeds that of G″, the system is dominated by elastic properties and typically exhibits gel-like characteristics. In the present study, the influence of particle concentration (*c*) and mass ratio (*k*) on the rheological behavior of Pickering HIPEs was systematically evaluated using amplitude sweep, frequency sweep, and shear rate sweep measurements (Figure 9).

In the stress sweep (1–100 Pa), all samples exhibited a clear linear viscoelastic region (LVER) at low stress levels, where both G′ and G″ remained stable and G′ consistently exceeded G″, indicating an elastic-dominant gel structure. As the applied stress increased, both moduli decreased significantly, suggesting progressive breakdown of the internal 3D network. With increasing particle concentration from 0.5% to 1.5%, the G′ value markedly increased and the decline in moduli became more gradual, indicating enhanced structural stability of the emulsion gel (Figure 9(A1)). This is attributed to higher particle coverage at the oil–water interface and increased spatial crowding and interlocking between droplets, leading to a denser 3D network [29]. Among systems with different β-CD/EGSC30 ratios (Figure 9(A2)), the *k* = 4:0 system showed the earliest modulus drop, whereas *k* = 3:1 and *k* = 2:2 exhibited more gradual changes, suggesting that composite particles formed a more stable gel network. This may be due to EGSC filling structural voids between β-CD particles and enhancing interparticle interactions via hydrogen bonding or physical cross-linking, thereby forming a more robust composite network [9].

To provide a more quantitative description, the LVER limit was defined as the stress at which G′ decreased by more than 5% from its plateau value. At a fixed β-CD/EGSC30 ratio of 3:1, the LVER limit increased from approximately 23.37 Pa at c = 0.5% to 48.39 Pa at c = 1.0% and 1.5%, indicating that increasing particle concentration enhanced the resistance of the emulsion gel network to deformation. At a fixed particle concentration of 1.5%, the k = 3:1 system showed the broadest LVER, with a limit of approximately 48.39 Pa, followed by k = 4:0 and k = 2:2, while the k = 1:3 system exhibited the narrowest LVER, with a limit of approximately 4.28 Pa. These results further demonstrate that an appropriate β-CD/EGSC30 ratio was essential for forming a stress-resistant HIPE network.

After determining the linear viscoelastic region (LVER), frequency sweep measurements were subsequently performed. Within the frequency range of 0.1–10 Hz, the storage modulus (G′) consistently exceeded the loss modulus (G″) for all samples, and no crossover point was observed. This result indicates a typical gel-like structure and suggests good mechanical stability of the systems. The dominance of elastic behavior is a common feature of Pickering HIPEs and is generally attributed to the close packing of emulsion droplets together with the interconnected network formed by particles adsorbed at the oil–water interface [30]. At fixed *k*, both G′ and G″ significantly increased with rising particle concentration (Figure 9(B1)), indicating enhanced viscoelasticity. This is because higher concentrations promote tighter interfacial particle packing and stronger droplet interactions, thus improving structural strength [31]. At constant concentration, different β-CD/EGSC30 ratios showed distinct modulus values (Figure 9(B2)), with k = 3:1 exhibiting the highest G′, followed by *k* = 2:2, while *k* = 1:3 and *k* = 4:0 showed lower values. This indicates that an appropriate amount of EGSC30 significantly enhances the viscoelasticity of the emulsion gel, likely due to its interfacial activity and ability to reinforce structural connections with β-CD. However, Excessive EGSC (k = 1:3) reduces the relative β-CD content at the oil–water interface, limiting interfacial coverage and particle–particle interactions, which weakens the overall structural strength of the Pickering HIPEs [8].

Figure 9(C1,C2) illustrate the variation in apparent viscosity of different systems as a function of shear rate. For all samples, the apparent viscosity decreased markedly as the shear rate increased, indicating pronounced shear-thinning behavior. This phenomenon can be attributed to the progressive disruption of the three-dimensional network structure and the rearrangement of emulsion droplets under increasing shear forces, which ultimately reduces internal resistance to flow [31]. At the same shear rate, apparent viscosity increased with particle concentration from 0.5% to 1.5% (Figure 9(C1)), consistent with amplitude and frequency sweep results, confirming that higher concentrations promote tighter droplet structuring and stronger networks. The viscosity trends across different β-CD/EGSC30 ratios mirrored those from frequency sweeps (Figure 9(C2)), with *k* = 3:1 showing the highest viscosity, while *k* = 4:0 and *k* = 1:3 exhibited lower viscosities and faster declines with shear, indicating greater susceptibility to shear-induced structural breakdown. These results further confirm a clear synergistic stabilization effect between β-CD and EGSC30, where an optimal ratio forms a more robust Pickering HIPE gel structure. The elastic-dominant structure and shear-thinning behavior of these Pickering HIPEs suggest their potential applicability in semi-solid high-oil food systems, such as high-oil dressings, spreads, structured fat replacers, and 3D-printable food pastes, where structural retention at rest and flowability under shear are both required.

## 4. Conclusions

In this study, esterified ginger starch (EGSC10, EGSC20, and EGSC30) with varying degrees of substitution (DSs) was successfully prepared via a dry-heat method using different concentrations of citric acid. The morphology, structure, and functional properties of the modified starches were systematically characterized by SEM, XRD, and FTIR. The results showed that esterification significantly reduced the relative crystallinity and short-range molecular order of starch. With increasing DSs, starch solubility and swelling power decreased, while resistant starch (RS) content markedly increased, indicating a structural reorganization into a more digestion-resistant architecture. Leveraging the interfacial activity of the modified particles, β-CD/EGSC30-based Pickering HIPE gels were successfully constructed. As particle concentration increased, droplet size decreased, and both gel strength and storage modulus (G′) were significantly enhanced. The system exhibited typical elastic-dominant and shear-thinning behavior, with the β-CD/EGSC30 ratio at 3:1 forming the most compact and stable 3D network. Overall, citric acid esterification enabled efficient construction of emulsion gels by synergistically modulating starch’s structural order and interfacial behavior, providing a theoretical foundation for the design of starch-based particle-stabilized emulsion systems.

## Figures and Tables

**Table 1:** The substitution degree, median diameter (D50), solubility and swelling power of GS and EGSC.

	Substitution Degree	D_50_ (μm)	Solubility (%)	Swelling Power (%)
GS	N.A.	10.90 ± 0.09 ^d^	8.97 ± 0.82 ^a^	6.86 ± 1.11 ^a^
EGSC10	0.060 ± 0.01 ^c^	12.86 ± 0.14 ^c^	5.82 ± 0.44 ^b^	4.90 ± 0.10 ^b^
EGSC20	0.088 ± 0.01 ^b^	14.21 ± 0.09 ^b^	2.76 ± 0.25 ^c^	3.74 ± 0.17 ^c^
EGSC30	0.110 ± 0.01 ^a^	20.48 ± 0.11 ^a^	1.38 ± 0.17 ^d^	2.97 ± 0.06 ^d^

Different lowercase letters in the same column indicate significant differences among samples (*p* < 0.05). N.A., data not determined.

**Table 2:** Resistant starch contents of GS and EGSC.

Sample	Resistant Starch Content	
	Before Cooking	After Cooking
GS	34.05 ± 0.51 ^c^	15.53 ± 0.71 ^c^
EGSC10	30.64 ± 0.57 ^d^	11.93 ± 0.13 ^d^
EGSC20	39.49 ± 0.29 ^b^	23.12 ± 1.08 ^b^
EGSC30	48.14 ± 0.15 ^a^	44.62 ± 0.18 ^a^

Different lowercase letters in the same column indicate significant differences among samples (*p* < 0.05).

## Data Availability

All the data is contained within the article, and the data presented in this study are available on request from the corresponding author.

## References

[1] Negi A., Barthwal R., Kathuria D., Singh N. (2024). Enzymatic advances in starch modification: Creating functional derivatives and exploring applications. Food Biosci..

[2] Lin X., Awais M., Zhu W., Raza H., Ren X. (2026). Physical modulation of starch/non-starch polysaccharide complexes for tailored functional foods: A comprehensive review. Carbohydr. Polym..

[3] Zhang M., Jia H., Wang B., Ma C., He F., Fan Q., Liu W. (2023). A Prospective Review on the Research Progress of Citric Acid Modified Starch. Foods.

[4] Cavallo E., Tupa Valencia M.V., Rossi E., Errea M.I., Foresti M.L. (2024). Production of resistant starches via citric acid modification: Effects of reaction conditions on chemical structure and final properties. Int. J. Biol. Macromol..

[5] Zheng H., Ma C., Bian X., Wang B., Xu Y., Zhang G., Liu X., Wang Y., Yang Y., Zhang N. (2026). Effects of rice starch esterified with citric acid and malic acid: Functional properties and structural characteristics. Carbohydr. Polym..

[6] Lee Y.-K., Chang Y.H. (2019). Structural and in vitro digestibility properties of esterified maca starch with citric acid and its application as an oil-in-water (O/W) pickering emulsion stabilizer. Int. J. Biol. Macromol..

[7] Zhou J., Tong J., Su X., Ren L. (2016). Hydrophobic starch nanocrystals preparations through crosslinking modification using citric acid. Int. J. Biol. Macromol..

[8] Chen H., Yang Y., Xu X. (2025). Citric acid esterification of rice starches from different sources for stabilizing Pickering emulsions. LWT.

[9] Li Y., Zhi Q., Liu Z., Bai L., Liu B. (2026). Construction and characterization of food-grade pickering high internal phase emulsions co-stabilized by β-cyclodextrin and konjac gum. Food Chem. X.

[10] Pan Y., Wang C., Geng S., Liu B. (2025). Ginger as a novel starch resource: Preparation, structural characterization and physicochemical properties. Food Chem..

[11] Lv X., Guo C., Ma Y., Liu B. (2022). Effect of citric acid esterification on the structure and physicochemical properties of tigernut starch. Int. J. Biol. Macromol..

[12] Argel-Pérez S., Gañán-Rojo P., Cuartas-Marulanda D., Gómez-Hoyos C., Velázquez-Cock J., Vélez-Acosta L., Zuluaga R., Serpa-Guerra A. (2023). Characterization of a Novel Starch Isolated from the Rhizome of Colombian Turmeric (*Curcuma longa* L.) Cultivars. Foods.

[13] Li S., He R., Liu J., Chen Y., Yang T., Pan K. (2024). Rod-Shaped Starch from Galanga: Physicochemical Properties, Fine Structure and In Vitro Digestibility. Foods.

[14] Schafranski K., Ito V.C., Lacerda L.G. (2021). Impacts and potential applications: A review of the modification of starches by heat-moisture treatment (HMT). Food Hydrocoll..

[15] Guo K., Lin L., Fan X., Zhang L., Wei C. (2018). Comparison of structural and functional properties of starches from five fruit kernels. Food Chem..

[16] Yang Q., Zhang W., Luo Y., Li J., Gao J., Yang P., Gao X., Feng B. (2019). Comparison of structural and physicochemical properties of starches from five coarse grains. Food Chem..

[17] Liu X., Qiao L., Kong Y., Wang H., Yang B. (2024). Characterization of the starch molecular structure of wheat varying in the content of resistant starch. Food Chem. X.

[18] Geng S., Wang Y., Liu B. (2024). Fabrication, characterization and application of Pickering emulsion gels stabilized by defatted grape seed powder. Food Chem. X.

[19] Geng S., Li Y., Lv J., Ma H., Liang G., Liu B. (2022). Fabrication of food-grade Pickering high internal phase emulsions (HIPEs) stabilized by a dihydromyricetin and lysozyme mixture. Food Chem..

[20] Remya R., Jyothi A.N., Sreekumar J. (2018). Effect of chemical modification with citric acid on the physicochemical properties and resistant starch formation in different starches. Carbohydr. Polym..

[21] Liu K., Yi J., Ma Y., Bi J. (2025). Esterification plays a significant role on apple pectin interaction with wheat starch: Impacting on gelatinization and gels properties. Food Hydrocoll..

[22] Arachchi D.M., Halim A., Fadimu G., Farahnaky A., Majzoobi M. (2025). Green Starch Modification Using Citric Acid: Quinoa, Chickpea, and Cassava Starches. Foods.

[23] Lu X., Xiao J., Huang Q. (2018). Pickering emulsions stabilized by media-milled starch particles. Food Res. Int..

[24] Low L.E., Siva S.P., Ho Y.K., Chan E.S., Tey B.T. (2020). Recent advances of characterization techniques for the formation, physical properties and stability of Pickering emulsion. Adv. Colloid Interface Sci..

[25] Zhao Q., Zaaboul F., Liu Y., Li J. (2020). Recent advances on protein-based Pickering high internal phase emulsions (Pickering HIPEs): Fabrication, characterization, and applications. Compr. Rev. Food Sci. Food Saf..

[26] Huang X.-N., Zhu J.-J., Xi Y.-K., Yin S.-W., Ngai T., Yang X.-Q. (2019). Protein-Based Pickering High Internal Phase Emulsions as Nutraceutical Vehicles of and the Template for Advanced Materials: A Perspective Paper. J. Agric. Food Chem..

[27] Wang Z., Zhao Y., Liu H., Chen Q., Liu Q., Kong B. (2024). Soy protein isolate-sodium alginate colloidal particles for improving the stability of high internal phase Pickering emulsions: Effects of mass ratios. Food Chem. X.

[28] Cui F., Zhao S., Guan X., McClements D.J., Liu X., Liu F., Ngai T. (2021). Polysaccharide-based Pickering emulsions: Formation, stabilization and applications. Food Hydrocoll..

[29] Dickinson E. (2015). Structuring of colloidal particles at interfaces and the relationship to food emulsion and foam stability. J. Colloid Interface Sci..

[30] Arditty S., Schmitt V., Giermanska-Kahn J., Leal-Calderon F. (2004). Materials based on solid-stabilized emulsions. J. Colloid Interface Sci..

[31] Dickinson E. (2012). Emulsion gels: The structuring of soft solids with protein-stabilized oil droplets. Food Hydrocoll..

